# A Nurse-Driven Protocol for Foley Catheter Utilization Decreases the Incidence of Traumatic Foley Catheterization

**DOI:** 10.31486/toj.20.0004

**Published:** 2021

**Authors:** Eric Laborde, Hayden Hill, Thomas E. Dukovac, Stephen P. Carriere, Kathleen Lata-Arias, Kristi Hebert, Raunak Patel, Jessie Gills

**Affiliations:** ^1^ Department of Urology, Ochsner Clinic Foundation, New Orleans, LA; ^2^ The University of Queensland Faculty of Medicine, Ochsner Clinical School, New Orleans, LA; ^3^ Crescent City Urology, New Orleans, LA; ^4^ Georgia Urology, Atlanta, GA; ^5^ OhioHealth Physician Group, Columbus, OH; ^6^ Department of Urology, Louisiana State University Health Sciences Center, New Orleans, LA

**Keywords:** *Catheter-related infections*, *hematuria*, *urethral catheterization*, *urinary catheters*, *urinary retention*, *urology*

## Abstract

**Background:** Traumatic urethral catheterization is a common reason for urologic consultation in hospitalized patients. The purpose of this study was to determine if a protocol designed to decrease Foley catheter use was effective and if implementation of the protocol decreased the incidence of Foley catheter–associated trauma.

**Methods:** In an effort to decrease catheter use, our institution adopted a nurse-driven Foley catheter protocol in May 2015 that allowed nurses to remove Foley catheters that did not meet criteria. We conducted a retrospective medical records review of patients who had Foley catheter–associated trauma occurring between February 2013 and March 2018 and compiled data concerning Foley catheter use. Using *t* test statistical analysis, we compared rates of Foley catheter use and Foley catheter–associated trauma before and after protocol implementation.

**Results:** During the 62-month study period, we documented 83 cases of Foley catheter–associated trauma. Prior to protocol implementation, our institution had mean of 2,903 patient-catheterization days per month. Following protocol implementation, the mean decreased to 2,604 patient-catheterization days per month (*P*<0.01). Prior to protocol implementation, the mean incidence of Foley catheter–associated trauma was 1.81 traumas per month. Following protocol implementation, the mean incidence decreased to 0.97 trauma per month (*P*<0.05).

**Conclusion:** Implementation of the protocol was successful in decreasing Foley catheter use as well as Foley catheter–associated trauma.

## INTRODUCTION

Traumatic urethral catheterization is a common reason for urologic consultation in hospitalized patients. Iatrogenic injury associated with urethral catheterization often presents as gross hematuria, severe pain in the penis or perineum, or lack of urine output via the Foley catheter. Such trauma can be a source of significant morbidity. Rarer complications include bladder rupture, perineal and bulbar artery pseudoaneurysm, intraperitoneal catheter misplacement via the verumontanum, autonomic dysreflexia in paralyzed patients, systemic infections, urethrovaginal fistula, and simultaneous bladder and small bowel perforation.^1-10^

Because of the focus on the risk of catheter-associated urinary tract infections (CAUTI) and on methods to reduce the incidence of this complication,^11-13^ many hospitals have implemented protocols to limit Foley catheterization days with the goal of decreasing CAUTI rates.^14,15^

In May 2015, our institution instituted a nurse-driven protocol for Foley removal (Appendix). In the protocol, approved indications for Foley catheterization included urinary retention, nonhealing sacral or perineal wound, required immobilization, hospice/comfort care, critically ill patient in the intensive care unit (ICU) requiring intensive monitoring, chronic indwelling Foley catheter on admission, or any Foley catheter placed or ordered by the urology service. These criteria limit the number of Foley catheters that can be placed. Per the protocol, nurses round daily and have a standing order to remove Foley catheters that do not meet the protocol criteria. Additionally, all physicians were educated about proper indications for Foley catheters, and Foley catheters could be ordered only if a patient met select criteria. We hypothesized that the incidence of traumatic Foley catheterization would decrease with the adoption of this protocol.

## METHODS

The urology service compiled a list of consultations for Foley catheter–associated traumas occurring between February 2013 and March 2018. Foley catheter–associated trauma was defined as urologic consultation for gross hematuria immediately following Foley catheter insertion or removal. After obtaining institutional review board approval, we conducted a retrospective medical records review of patients who had Foley catheter–associated trauma during the study period. Data collected included patient age, date of consultation, interventions required, complications, indication for placement, and use of blood thinning medications. Data were also collected on Foley catheter use for all hospitalized patients during the study period.

We compared rates of Foley catheter–associated trauma prior to protocol implementation to the rates after protocol implementation and assessed the rate of traumas per month. We also assessed the number of patient-catheterized days per month before protocol implementation and compared them to patient-catheterized days per month after protocol implementation using a 2-tailed *t* test. SPSS software, version 21.0 (IBM Corp) was used for statistical analysis.

## RESULTS

During the 62-month study period, we documented 83 cases of trauma secondary to Foley catheterization. In 68 of 83 (82%) cases, the trauma occurred during catheter insertion, and in 15 of 83 cases (18%), the trauma occurred when patients removed the catheter with the balloon inflated. Twenty-three patients (28%) required procedural or operative intervention for their trauma. Demographically, patient age, race, and use of blood-thinning medications were similar before and after implementation of the protocol.

Prior to protocol implementation, 49 cases of Foley catheter–associated trauma occurred in 27 months, for a mean incidence of 1.81 traumas per month. Following protocol implementation, 34 cases of Foley catheter–associated trauma occurred in 35 months, for a mean incidence of 0.97 trauma per month—a 46% reduction in incidence (*P*<0.05).

During the 27 months of the study period prior to protocol implementation, the number of patient-catherization days at our institution was 78,380 days, for a mean of 2,903 patient-catheterization days per month. During the 35 months of the study period following protocol implementation, the number of patient-catherization days at our institution was 91,140 days, for a mean of 2,604 patient-catheterization days per month (*P*<0.01).

## DISCUSSION

Foley catheter–associated trauma is a source of morbidity for patients and historically has been fairly common, with a study from 2008 citing a rate of 3.2 injuries per 1,000 male patient admissions.^16^ This morbidity is often iatrogenic and can be prevented with proper insertion techniques and by avoiding unnecessary catheterization. Sullivan et al reported that the most common complication of urethral catheterization in their study population was iatrogenic urethral injury, accounting for 55% of catheter complications.^17^ Importantly, Sullivan et al reported that for patients who sustained a complication from urethral catheterization, monitoring urinary output was the most common indication cited for urethral catheterization. Routine urinary output monitoring is no longer an acceptable indication for Foley catheterization at our institution. Therefore, avoiding unnecessary urethral catheterization can prevent a large proportion of complications from catheterization, especially urethral injury.

The added morbidity from such complications can lead to the need for surgery and increased length of stay that can significantly increase hospital costs. Davis et al found that patients who sustained an iatrogenic urethral catheter injury had additional costs of $10,000 per hospitalization.^18^ Costs included ICU stay, extended hospitalization, bedside and surgical interventions, catheters, and wires. Iatrogenic urethral catheterization complication increased length of stay by 9.4 ± 10 days (range, 2 to 53 days). Davis et al noted that these costs were likely underestimated, as they did not include costs associated with long-term complications, repeat interventions, and follow-up appointments. These results demonstrate that decreasing Foley catheter–associated trauma can improve patient outcomes and reduce the cost of health care.

After implementation of our protocol, the rate of Foley catheter–associated trauma per month decreased. Foley catheter use similarly decreased following implementation of the protocol, supporting the idea that the decrease in the Foley catheter–associated trauma rate is likely related to the decreased utilization. Utilization also highlights that areas for future improvement could focus on making each urethral catheter insertion event safer, possibly by improving the training of nurses and house staff or by focusing on decreasing the inherent risk of the catheters.

A limitation of this study is that we relied on consultations to define Foley catheter–associated trauma. Trauma could have occurred without a consult to the urology service, so all occurrences of trauma may not have been captured.

## CONCLUSION

A protocol designed to decrease Foley catheterization not only significantly decreased the mean number of patient-catherization days per month, but also significantly reduced Foley catheter–associated trauma. We encourage other hospitals to adopt similar protocols.

## Notes

This article meets the Accreditation Council for Graduate Medical Education and the American Board of Medical Specialties Maintenance of Certification competencies for Patient Care, Medical Knowledge, and Practice-Based Learning and Improvement.
